# Acorn flour from holm oak (*Quercus rotundifolia*): Assessment of nutritional, phenolic, and technological profile

**DOI:** 10.1016/j.crfs.2022.11.003

**Published:** 2022-11-04

**Authors:** Rita Beltrão Martins, Irene Gouvinhas, Maria Cristiana Nunes, Luís Mendes Ferreira, José A. Peres, Anabela Raymundo, Ana I.R.N.A. Barros

**Affiliations:** a CITAB - Centre for the Research and Technology of Agro-Environmental and Biological Sciences/ Inov4Agro - Institute for Innovation, Capacity Building, and Sustainability of Agri-Food Production, Universidade de Trás-os-Montes e Alto Douro; Quinta de Prados, 5000-801, Vila Real, Portugal; b CQVR- Chemistry Centre of Vila Real, Universidade de Trás-os-Montes e Alto Douro, 5000-801, Vila Real, Portugal; c LEAF - Linking Landscape, Environment, Agriculture and Food, Instituto Superior de Agronomia, Universidade de Lisboa, Tapada da Ajuda, 1349-017, Lisbon, Portugal

**Keywords:** Underexploited resource, Acorn flour, Gluten-free flour, Fiber-rich, Unsaturated fatty acids, Antioxidant capacity

## Abstract

Acorn is the fruit of holm oak (*Quercus rotundifolia*), being mainly used nowadays to feed animals, however a substantial part remains in the fields without any valorization. Underexploited crops are gaining new interest, driven by food security concerns and health benefits potential as well. In the present work, it was studied the physicochemical characteristics and functional perspective of acorn flour, as an ingredient for human diet. The study included nutritional composition analysis, phenolic compounds profile through HPLC, starch content and its microstructure, fibre, and pasting properties assessment. Acorn flour presented a high content in fat, particularly monounsaturated and polyunsaturated (oleic and linoleic acids), and high minerals content in particular K. Concerning phenolic profile, rutin, catechin, ellagic acid, gallic acid, and syringic acid were identified. In regards to technological profile, fibre was mainly insoluble, with around 11%, and starch content was 50%. Its pasting behaviour revealed a high gelatinization temperature (85 °C), with low breakdown, and higher retrogradation consistency. These results show acorn flour potential as a valuable and sustainable multipurpose food ingredient.

## Highlights

• Acorn flour (*Quercus rotundifolia*) was chemical and technologically assessed.
• Acorn flour revealed high content in unsaturated fatty acids (oleic and linoleic).
• Minerals were analysed for the first time, revealing high values in particular potassium.
• Phenolic acids and flavonoids were identified in particular rutin and syringic acid.
• Pasting behaviour has shown high gelatinization temperature and shear stress resistance.

## Graphical abstract

**Figure:**
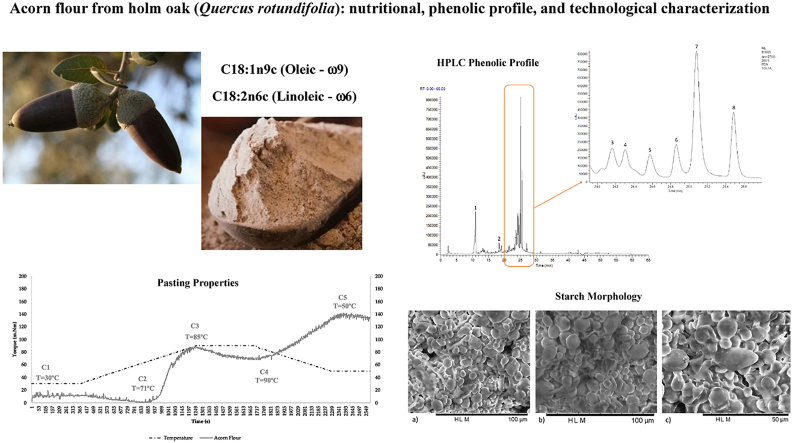

## 1 Introduction

Acorn is the fruit of oak, an evergreen tree from *Quercus* genus, which belongs to the *Fagaceae* family. In particular, holm oak (*Quercus rotundifolia*) is one of the most important trees in the unique Mediterranean woodland ecosystem named “Montado | Dehesa” (Silva, 2007). To better understand the given names to holm oak acorn, it is important to highlight that *Quercus rotundifolia* is also identified as *Quercus ilex* subsp. *Ballota*, or also *Quercus ilex* subsp. *rotundifolia*. The three names mean the same tree, nevertheless in the last years, *Quercus rotundifolia* and *Quercus ilex* have been considered two different species (Silva, 2007). In addition, other authors have been demonstrating the separation of both species, referring that *Quercus rotundifolia* is a different species from *Quercus ilex,* not only for its different physicochemical characteristics, but also due to the geographical distribution: Southwest of Iberian Peninsula for *Quercus rotundifolia*, and Northeast of Iberian Peninsula, south of France and Italy for *Quercus ilex* (Rafii et al., 1991; Silva, 2007)*.*

Historically, during Roman Era and even before, there are many references to the use of acorns and acorn flour in the Mediterranean region. Acorns were an important source of food, in particular during hunger periods, in bad harvesting years (Jacob, 2003). However, its regular consume was lost, and nowadays acorns are mainly consumed by grazing animals, in particular Iberian pigs breed, but still a great part is left in the fields without any valorization. Additionally, acorns from South of Europe have the particular characteristic of being sweet, due to lower tannins content, when compared with other oak species, which makes even more interesting to be used as a common ingredient in human diet (Vinha et al., 2020). Moreover, holm oak is a protected tree that is forbidden to cut, which accordingly to European law its implementation areas cannot be used to any other purpose, thus, it is really important to give a use to this valuable resource. The last forest inventory in 2015, revealed that in Portugal exists approximately 350 000 ha of holm oak trees, which is a large area, in particular comparing with a high valuable crop as chestnut tree for example, which the total estimated area is much lower, about 48 000 ha (National Forest Inventory - Portugal, 2019).

In the last decade, there has been a growing interest in the use of unconventional crops as potential ingredients to incorporate in human diet in a daily basis. Not only due to the expected food scarcity, but also owing to the well-known health potential of these resources (Santos, Lopes da Silva and Pintado, 2022). Acorn has not been an exception, and many studies highlight its characteristics (Vinha et al., 2016, Vinha et al., 2016; Vinha et al., 2020).

Acorn flour seems to be an easy way of recovering the use of acorn in human diet, not only in bakery products, but also for many other purposes (Correia, Nunes, & Beirão-da-Costa, 2013; Vinha et al., 2016). Acorn flour is naturally gluten-free (GF) and presents an interesting nutritional and functional profile. It is rich in unsaturated fatty acids and fiber, minerals, vitamin A and E, chlorophylls, carotenoids, other polyphenols, and present high antioxidant capacity (Silva et al., 2016; Vinha et al., 2016). Many authors have been studying acorn bioactive compounds and antioxidant capacity, being undoubtedly one of the most interesting characteristics of this fruit (Cantos et al., 2003; Tejerina et al., 2011; Vinha et al., 2016). Specifically, extracts from holm oak acorn, revealed several important health benefits (reviewed in Vinha et al., 2020).

From a baking point of view, acorn flour seems to be very interesting not only due to its baking characteristics, contributing to gluten replacement role, but also to improve nutritional properties of GF bread, and consequently supporting a balanced GF diet. Many studies have presented promising results in using acorn flour to develop baking products, both gluten-free and gluten containing, such as bread (Beltrão Martins et al., 2020; Korus et al., 2015; Martins et al., 2020; Skendi et al., 2018), particularly GF bread in combination with sourdough (Beltrão Martins et al., 2022), cake, and biscuits (Pasqualone, Roma and Acciani, 2019; Korus et al., 2017; Molavi et al., 2015). As a baking ingredient, acorn flour physicochemical characteristics are really important. It gains even more relevance, if acorn flour is used in GF baking, where gluten replacement is quite challenging, and starch behaviour is of great importance, since it is the main structural ingredient in the bread (Matos and Rosell, 2015). On the other hand, fiber content is also relevant, due to its influence in terms of structure and nutrition (Föste et al., 2020). In addition, many other products could be developed with acorn, in particular functional foods, pharmaceutical, and cosmetic formulations (Vinha et al., 2020).

Several authors have been studying acorn flour in many different aspects, and besides the variability among the different acorn species, to the best of our knowledge, *Quercus rotundifolia* has not been studied yet in both chemical characteristics and technological performance perspectives. Thus, the aim of the present study was to characterize acorn flour as a common ingredient for human diet, in a physicochemical and functional perspective, including nutritional, fatty acids, and minerals composition, phenolic profile, and also in a technological view, analyzing its starch microstructure by SEM, pasting properties and fiber content.

## 2 Materials and methods

### 2.1 Reagents

Reagents manufacturers: sodium nitrite, aluminum chloride, and sodium hydroxide, all extra pure (>99%), saline water (0.9% NaCl), and methanol were acquired from Merck (Merck, Darmstadt, Germany). Folin–Ciocalteu's reagent, 3,4,5-trihydroxybenzoic acid (gallic acid), acetic acid, both extra pure (>99%), and sodium hydroxide were purchased from Panreac (Panreac Química S.L.U., Barcelona, Spain). Sodium molybdate (99.5%) was obtained from Chem-Lab (Chem-Lab N.V., Zedelgem, Belgium). The compounds 2,2-azino-bis(3-ethylbenzothiazoline-6-sulphonic acid) diammonium salt (ABTS+), 2,2-diphenyl-1-picrylhidrazyl radical (DPPH), potassium phosphate, catechin, potassium persulfate, sodium acetate, 2,4,6-Tripyridyl-s-Triazine (TPTZ iron reagent), acetic acid, hydrochloric acid, and iron(III) chloride were obtained from Sigma-Aldrich (Sigma-Aldrich Produktions GmbH, Steinheim, Germany). Additionally, 6-hydroxy-2,5,7,8-tetramethylchroman-2-carboxylic acid (Trolox) was purchased from Fluka Chemika (Fluka Chemika, Neu-Ulm, Switzerland). Ethanol, ether petroleum, potassium persulfate, sodium hydroxide, methanol, hydrochloric, formic, and nitric acid, acetonitrile, iron(III) chloride, sodium acetate, sodium carbonate, sulphuric acid (98%), and potassium hydroxide (98%) were acquired from Panreac (Panreac Química S.L.U., Barcelona, Spain).

### 2.2 Acorn flour sampling

Acorn flour obtained from holm oak (*Quercus rotundifolia*) was sampled from 2.5 kg buckets available in the market (Terrius, Marvão, Portugal). Samples were collected from ten different buckets, from two different batches. Batches were correspondent to different consequent years (2018 and 2019), and acorns were harvested in two different geographical areas in the South of Portugal (Évora and Portalegre). The acorn flour in study is produced from *Quercus rotundifolia* specie acorns. In order to protect the flour from oxidation, smaller packages were prepared in food safe plastic bags and closed under vacuum.

### 2.3 Acorn flour chemical composition determination

Acorn flour chemical composition was performed according to the Association of Official Analytical Chemists methods (AOAC, 2016) for moisture, ash, protein, fat and carbohydrates content. All nutritional analyses were carried out in triplicate and the results presented as mean value and respective standard deviation.

Moisture Content was assessed using an automatic moisture analyser PMB 202 (Adam Equipment, Oxford, USA), through gravimetric measurements, after reaching constant weight at 130 °C. Ash Content was measured gravimetrically, incinerating samples at 550 °C in a muffle furnace L 9/R (Nabertherm, Lilienthal, Germany). Fat content was determined by using petroleum ether in a Soxhlet extractor Det-gras N (JP Selecta, Barcelona, Spain), dried in the oven (overnight), and finally fat content was calculated gravimetrically. Protein content was assessed using Kjeldhal method (Nx6.25). Carbohydrates were determined by difference (100 - % moisture - % ash - % protein - %fat).

### 2.4 Fatty acids profile determination

After previously determination of fat content, according to the aforementioned method, fatty acids profile was determined using the method ISO 5509:2000, with some modifications. Finally, methyl esters were prepared and then analysed in a GC-FID Agilent 7820A (Agilent, Santa Clara, CA, USA). Analysis were carried out in triplicate.

### 2.5 Acorn flour mineral analysis by ICP

Mineral content was determined using acid digestion followed by absorbance reading in an Inductively Coupled Plasma Optical-Emission Spectrometry (ICP-OES) Thermo ScientificTM iCap Series 7000 (Thermo Fisher Scientific, Waltham, MA, USA). A sample of 0.25 g was digested with a mixture of nitric and hydrochloric acid (ratio 1:3) at 105 °C. The mixture was cooled down and when room temperature was reached, it was filtered and diluted to 50 mL with distilled water. The extracts were then analysed for K, Ca, Mg, P, S, Fe, Zn, and Mn. Analysis were carried out in triplicate.

### 2.6 Bioactive compounds and antioxidant capacity determination

Bioactive compounds extraction from acorn flour samples was performed weighting 40 mg of sample, and adding 1.5 mL of extracting solvent (methanol/distilled water (70:30, v/v)) (R Beltrão Martins et al., 2020). Samples were stirred at highest speed, for 30 min, at room temperature, and then centrifuged at 5000 rpm for 15 min at 4 °C. Supernatant was collected. Final volume was made up to 5 mL with methanol/distilled water (70:30, v/v). The procedure was repeated three times.

#### 2.6.1 Determination of total phenols content, *ortho*-diphenols, and flavonoids

To measure total phenols content in acorn flour, Folin-Ciocalteu reagent was used, with gallic acid as standard. This method consists in the phosphowolframate–phosphomolybdate complex reduction through acorn flour's phenolic compounds, into blue reaction products. Briefly, 20 μL of sample and 100 μL of previously diluted Folin-Ciocalteu reagent were mixed and vortexed. Then, 80 μL of sodium carbonate were added and mixed in a vortex. Finally, after 30 min of incubation in the oven at 40–45 °C in the dark, the absorbance was read at 750 nm. Results were expressed in milligrams of gallic acid per gram of dry weight (mg GA g^−1^ DW) (Gouvinhas et al., 2018).

*Ortho*-diphenols in acorn flour samples were measured using the protocol as previously described in Gouvinhas et al. (2018). 40 μL of sodium molybdate were added to 160 μL of samples properly diluted. Mixtures were vortexed and protected from light during 15 min, at room temperature, before reading absorbance at 375 nm. *Ortho*-diphenols content was quantified using gallic acid as standard, and results expressed in milligrams of gallic acid per gram of dry weight (mg GA g^−1^ DW).

Acorn flour flavonoids were assessed through the following experimental procedure: 24 μL of sample were mixed with 28 μL of sodium nitrite (50 g L^−1^). After waiting 5 min, 28 μL of aluminum chloride (100 g L^−1^) were added and the mixture reacted for 6 min. Next, 120 μL of sodium hydroxide (1 M) were added to the mixture, the microplate was shaken for 30 s, and absorbance measured at 510 nm. Flavonoid content was quantified using catechin as standard, and results presented in milligrams of catechin per gram of dry weight (mg CAT g^−1^ DW) (Gouvinhas et al., 2018).

#### 2.6.2 Determination of antioxidant capacity

ABTS radical inhibition was assessed with the following procedure: a sample (12 μL) or standard (12 μL) were poured into the microplate, and then previously prepared ABTS working solution (188 μL) was added. Next, the reaction was completed at room temperature, for 30 min, in the dark, and absorbance measured at 734 nm. ABTS^•+^ radicals inhibition was determined through the expression: $\%inhibition=\left(\frac{{Abs}_{blank}-{Abs}_{sample}}{{Abs}_{blank}}\right)\times 100$.

Acorn flour extracts antioxidant activity was calculated by Trolox calibration curve interpolation, and the results were expressed in mmol of Trolox per gram of dry weight (mmol Trolox g^−1^ DW).

DPPH antioxidant capacity was performed throughout adding in each well of the microplate, the sample (10 μL) or Trolox standard (10 μL), and DPPH (190 μL). Next, the incubation occurred in the dark, at room temperature for 30 min, and the absorbance was read at 520 nm. Free radical DPPH^•^ inhibition was determined using ABTS^•+^ expression above referred. Acorn flour samples scavenging capacity was determined by Trolox calibration curve interpolation, and the results were expressed in mmol of Trolox per gram of dry weight (mmol Trolox g^−1^ DW).

Ferric reducing antioxidant power (FRAP) was measured following the steps: the sample (20 μL) was placed in the microplate, and previously prepared FRAP working solution (280 μL) was added. Then, the reaction was performed in the dark, for 30 min at 37 °C, and mixing at 250 rpm, with an incubator PST-100HL (BioSan, Riga, Latvia), and finally the absorbance was read at 593 nm. The standard was Trolox, and results were expressed in mmol of Trolox per gram of dry weight (mmol Trolox g^−1^ DW).

#### 2.6.3 Qualitative and quantitative assessment of phenolic compounds

The phenolic profile of acorn flour samples was assessed as described in (Leal et al., 2020) using a Reverse Phase–High-Performance Liquid Chromatography–Diode Array Detector (RP-HPLC-DAD), with a C18 column (4.6–250 mm, 5 μm particle size; ACE, Aberdeen, Scotland). The method of reverse phase HPLC is based in a polar mobile phase with a mixture of solvent A, H_2_O/HCOOH (99.9:0.1, v/v), and solvent B, CH_3_CN/HCOOH (99.9:0.1, v/v), together with a non-polar stationary phase. A linear gradient scheme was used, with the following characteristics: (min; %B): (0; 5%), (15; 15%), (30; 30%), (40; 50%), (45; 95%), (50; 95%), and (55; 5%). At 55 min, return to 5% of B to stabilize and prepare the column for the next sample. All of the analysis was performed at room temperature (25 °C) with a flow rate of 1.0 mL min^−1^. The injection volume of the samples was 20 μL. All samples were injected in triplicate. The equipment consisted of an LC pump (SRVYR-LPUMP), an auto-sampler (SRVYR-AS), and a photodiode array detector (SRVYR-PDA5) in series (Thermo Fisher Scientific, Inc., Waltham, MA, USA).

All the compounds were identified at 280 nm using the standards of: gallic acid, catechin, syringic acid, ellagic acid, rutin, and luteolin. For the quantification of unidentified flavanols, the standard of rutin was used. The results were expressed in milligrams per gram of dry weight (mg g^−1^ DW) (Leal et al., 2020).

### 2.7 Water binding capacity determination

The acorn flour water binding capacity (WBC) determination was performed according to AACC (1999), as described in Espinosa-Ramírez et al. (2018). Briefly, acorn flour samples were weighted, the water was added, well mixed and centrifuged (Thermo Scientific, Waltham, Ma, EUA) at 2000×*g* for 10 min. Finally, supernatant water was discharged, weighed again, and by gravimetrically difference, WBC was calculated. Results were expressed in g of water per g sample (g water/g sample) (Espinosa-Ramírez et al., 2018).

### 2.8 Total fiber quantification

Acorn flour total fiber (TF) and insoluble fiber (IF) content were determined by using a “Total Dietary Fiber Assay Kit” from Megazyme (Wicklow, Ireland), following manufacture instructions. All procedures were done at least in duplicate. Soluble fiber (SF) was calculated by difference (SF = TF-IF).

### 2.9 Total starch quantification and SEM morphological characterization

Acorn flour starch content was determined using an enzymatic kit K-TSTA-100A (Megazyme, Enterprise Ireland, Dublin, Ireland), and then was measured in a spectrometer UVmin-1240 (Shimadzu, Kyoto, Japan).

Starch microstructural characterization was carried out with a scanning electron microscopy – SEM (Hitachi SEM TM 3030Plus, Tokyo, Japan). Samples were observed with 400–1500x magnification.

### 2.10 Acorn flour pasting properties

The acorn flour pasting properties were performed using a Micro-doughLAB 2800 (Perten Instruments, Sidney, Australia), following the manufacturer's cooking protocol with some modifications (Dang and Bason, 2015). Constant mixing rate at 63 rpm for 43 min (2580 s), and the following temperature cycle: 30 °C for 6 min, then rise temperature until 90 °C during 15 min, the temperature of 90 °C keeps constant for 7 min, then decrease to 50 °C during 10 min and finally, constant temperature of 50 °C for more 5 min, until the end. 4.00 ± 0.01 g of acorn flour sample was weighed and placed in the chamber of Micro-doughLAB. The amount of water added was in accordance to flour's moisture (8.15%), for a water absorption of 65% and 14% moisture basis. Tests were performed at least in triplicate.

## 3 Results & discussion

### 3.1 Acorn flour chemical composition

The obtained values (in g 100 g^−1^) for acorn flour nutritional composition were: moisture 8.15 ± 0.03, ash 1.61 ± 0.01, total protein 4.28 ± 0.27, total fat 11.39 ± 0.53, and carbohydrates 74.56 ± 0.76. These results were in agreement with the values range obtained for proximal composition, in a study about variability between acorns from nine different trees, also *Quercus rotundifolia*, which were (in g 100 g^−1^): ash (1.37–1.76), protein (3.84–6.09), and fat (9.34–13.00) (López-Hidalgo et al., 2021).

Acorn flour presented the highest fat content when comparing with other common flours (11.39 g 100 g^−1^), such as rice (0.90), wheat (1.81), maize (2.48), sorghum (3.50), wholewheat (3.63), buckwheat (4.21), or oat (6.74). Concerning protein (4.28 g 100 g^−1^), acorn flour revealed a lower value than quinoa (13.48), buckwheat (12.19), wheat (11.54) and rice flour (7.33), but similar to sorghum (4.68), and higher than cassava (1.7) (Hager et al., 2012). Protein amount has influence not only in nutritional profile, but also in technological performance of acorn flour, which despite its lower value, in comparison with other flour sources, has a higher fat content. Acorn's high fat content it is well known and can be used with advantage as a food ingredient due to fat characteristics as a texture improver, namely resulting from high unsaturated fatty acids. On the other hand, also health benefits can be seen as an advantage, since acorn fat is mainly unsaturated (next section).

### 3.2 Acorn flour fatty acids profile and minerals

Fatty acids profile of acorn flour is shown in Table 1. Acorn flour revealed oleic acid as the main fatty acid in its composition, approximately 65%, followed by linoleic acid, being almost 15%, next to palmitic acid with about 14%, and finally stearic acid with nearly 4%. With much lower values, were eicosonoic, eicosenoic, α-linolenic acids, with respectively 0.54%, 0.64%, and 0.51%. These results were quite similar to those found in other studies about acorn flour fatty acid profile (López-Hidalgo et al., 2021; Tejerina et al., 2011). López-Hidalgo et al. (2021) obtained a range of values from the nine studied trees, which were: oleic acid from 59.57% to 63.57%, linoleic acid from 12.98% to 17.22%, palmitic acid, from 16.15% to 20.32%, and stearic acid from 2.94% to 3.83%, but did not mention the acids with lower concentrations. Tejerina et al. (2011) found, as annual average of two different years 2007 and 2008 respectively, the following results: oleic 61.84%; 62.95%, linoleic 17.80%; 17.67%, palmitic 13.24%; 13.37%, stearic 3.04%; 3.46%, eicosonoic 0.38–0.46, eicosenoic 0.53–0.57%, α-linolenic 0.95–0.88%. The closeness of the results is an important aspect, in order to understand acorn's fatty acids profile, since many authors highlight a general variability between these fruits. Nevertheless, comparing the same species, it was possible to find approximately results, concerning the ratios of fatty acids profile.

**Table 1:** **–** Acorn flour fatty acids and minerals profile.

Fatty acids (g 100 g^**−1**^ of Fat)		Minerals (mg 100 g^**−1**^ DW)	
C16:0 (Palmitic)	14.11 ± 0.50		
C18:0 (Stearic)	3.68 ± 0.02	K	697.10 ± 6.02
C20:0 (Eicosonoic)	0.54 ± 0.08	Ca	51.66 ± 1.84
C18:1n9c (Oleic - ω9)	66.26 ± 0.98	Mg	65.82 ± 1.62
C18:2n6c (Linoleic - ω6)	14.72 ± 0.08	P	81.56 ± 1.39
C20:1 (Eicosenoic)	0.64 ± 0.02	S	57.15 ± 0.54
C18:3n3 (Linolenic ω3)	0.51 ± 0.04	Fe	0.81 ± 0.05
Saturated	18.71 ± 0.49	Mn	7.78 ± 0.24
Monounsaturated	67.48 ± 0.47	Zn	0.54 ± 0.00
Polyunsaturated	14.72 ± 0.08		

Results presented as mean value and respective standard deviation.

Thus, acorn flour revealed a higher proportion of monounsaturated fatty acids (MUFA), mainly oleic, and in polyunsaturated fatty acids (PUFA), linoleic and α-linolenic, which are essential fatty acids. Both group of fatty acids, presents many benefits from an health point of view, such as preventing the risk of cardiovascular diseases, lowering blood serum triglycerides, and increasing HDL-cholesterol (high density lipoprotein cholesterol) levels, among others. Moreover, regarding functional advantages, acorn also presents β-sitosterol as the predominant phytosterol, and is an important source of tocopherols, exhibiting as well a profile quite similar to olive oil (reviwed by Taib et al., 2020).

Concerning minerals, represented in Table 1, it is possible to understand that K was the most abundant mineral in acorn flour, with almost 700 mg 100 g^−1^, which is a very high amount, followed by P, Mg, S, Ca, and finally the microelements Na, Mn, and Fe. As far as we are aware of, *Quercus rotundifolia* acorn mineral profile, has not been published before, so is not possible to compare our results. However, values from *Quercus robur* acorn are available, with the following results: 0.83% K, 0.1% Ca, 0.1% P, 0.04% Mg, and also microelements (mg kg^−1^) 41.00 Fe, 3.00 Mn, 6.5 Zn (Rakić et al., 2006). Thus, it is possible to establish a comparison with the results from the present work, although the difference in species, which converting for the same units, were: 0.70% K, 0.05% Ca, 0.08% P, 0.07% Mg, and concerning microelements (mg kg^−1^), 8.1 Fe, 77.8 Mn, and 5.40 Zn. Thus, K, and P revealed quite similar results, Ca was lower, and Mg was higher, in comparison with *Quercus robur* acorn. Fe presented a really lower value, Mn was much higher, and Zn was similar. *Quercus rotundifolia* acorn flour showed to be a good source of minerals, in particular K, even when comparing with common flour sources such as wheat, oat or sorghum, which usually present lower values of these minerals. This is a relevant aspect, because potassium has an important role in muscles, and minerals in general are essential for human health, since take part in many metabolic processes (Dias, 2021).

### 3.3 Bioactive compounds and antioxidant capacity

The obtained values for acorn flour phenolic composition were 16.79 ± 0.43 for total phenols, 33.61 ± 0.72 for *Ortho*-diphenols, and 3.20 ± 0.26 for flavonoids content. Concerning antioxidant activity, the results were respectively, 0.51 ± 0.04, 0.27 ± 0.01, and 0.36 ± 0.04, for ABTS, DPPH, and FRAP methodologies. Taking in consideration the limitations of comparing bioactive compounds results, and its respective antioxidant capacity due to extraction and methodologies specifications (Monteiro et al., 2020), it was possible to make comparisons with Vinha et al. (2016) and Tejerina et al. (2011). These authors, obtained quite similar values (in mg GA g^−1^ DW), respectively 18.0 and 14.3 for acorn flour phenolic compounds (Tejerina et al., 2011; Vinha et al., 2016).

Comparing the results from the present study with other ingredients, in a research about several GF flours polyphenols characterization, black rice revealed the highest value, with 1.48 mg GAE g^−1^ (Rocchetti et al., 2017). Regarding our results it is possible to understand that acorn flour revealed 10 times higher total phenols content than black rice.

### 3.4 Qualitative and quantitative phenolic profile assessment

**Fig. 1:**
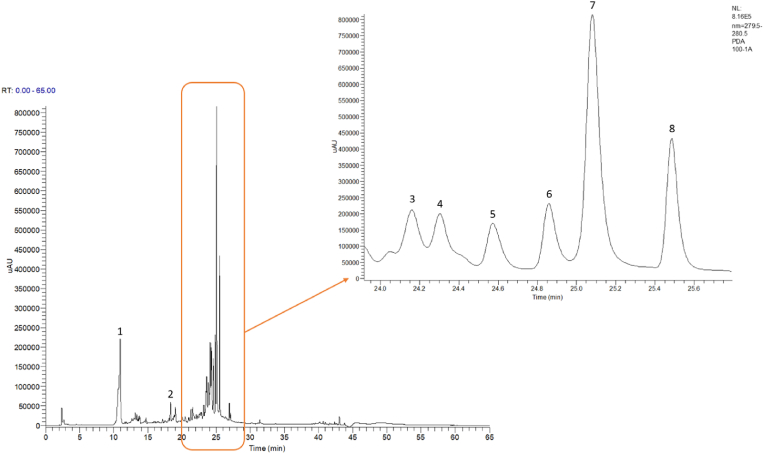
**–** Acorn flour phenolic profile with a zoom representation between 24.0 and 25.0 min of retention time recorded at 280 nm.

In Fig. 1 acorn flour phenolic profile is depicted, with the correspondingly identification of each compound in Table 2.

**Table 2:** Phenolic compounds identified in acorn flour, by retention time and expressed in mg g^−1^ DW.

ID number*	Identification	RT* (min)	Concentration (mg g^−1^ DW)
1	Gallic acid	10.90	13.27
2	Catechin	18.35	6.11
3	Syringic acid	24.16	21.23
4	Benzoic acid unidentified	24.33	5.22
5	Ellagic acid	24.57	5.05
6	Rutin	24.86	16.29
7	Flavanol unidentified	25.08	149.15
8	Flavanol unidentified	25.49	65.50

Results presented as mean value and respective standard deviation. ID number = identification number, RT = retention time.

There were found eight compounds belonging to the classes of phenolic acids and flavonoids. The compounds identified were gallic acid, catechin, syringic acid, ellagic acid, and rutin with respectively concentrations of 13.27; 6.11; 21.23; 5.05; 16.29 mg g^−1^ DW. Additionally, was recognized a benzoic acid structure and two flavanols that were not possible to identify, which concentrations were respectively, 5.22; 149.15; 65.50 mg g^−1^ DW.

Cantos et al. (2003) determined phenolic composition in acorn (*Quercus rotundifolia*), performing similar extracts to the ones in the present work, with methanol/water 80:20 (v/v). These authors found gallic acid, ellagic acid, and their derivatives. Moreover, it was also found several compounds that could not be identified in our samples, which were galloyl glucoside, valoneic acid dilactone, and their derivatives (Cantos et al., 2003). Despite not being identified in the latter reference, rutin, catechin, and syringic acid, were found in other acorn species (reviewed in Vinha et al., 2016).

### 3.5 Total fiber and starch content

In Table 3 it is possible to observe acorn water binding capacity (WBC), total, insoluble and soluble fiber, and total starch content of acorn flour. Water binding capacity (WBC) is defined as the amount of water retained by the flour structure (namely starch, fiber, protein, and its interactions) after centrifugation. This is a fundamental parameter for baking formulation, since the amount of water that the flour can bind will have a major role in the development of the dough, and consequently in the final product quality. WBC obtained for acorn flour was 1.19 ± 0.06 g water/g sample, which in comparison with other flour sources, such as rice (1.38 g water/g sample), is an inferior value. This was expected, due to the lower starch content in acorn flour, in comparison with rice flour (80–90 g 100 g^−1^ DW). Consequently, acorn flour could contribute to a reduction of the WBC when mixed with other flours, nevertheless the complexity of food matrices, along with the interactions of starch, fiber, protein, fat, and water, does not allow to establish a direct cause-effect answer.

**Table 3:** Water binding capacity (WBC), total, insoluble and soluble fiber, and total starch content of acorn flour.

WBC (g H_2_O/g sample)	Total Fiber (g 100 g^−1^ DW)	Insoluble Fiber (g 100 g^−1^ DW)	Soluble Fiber (g 100 g^−1^ DW)	Total Starch (g 100 g^−1^ DW)
1.19 ± 0.06	11.40 ± 1.56	10.30 ± 1.13	1.10 ± 0.42	50.90 ± 2.40

Results presented as mean value and respective standard deviation.

Concerning total fiber, acorn flour revealed an amount of 11.40 g 100 g^−1^ DW and 10.30 ± 1.13 g 100 g^−1^ DW for insoluble fiber. Silva et al. (2016) obtained a higher fiber content 17.90 ± 2.95 g 100 g^−1^ DW and was quite lower (2.07–2.25 g 100 g^−1^) in López-Hidalgo et al. (2021), showing the variability previously mentioned. Acorn flour's fiber content was similar to wholewheat, and higher than the majority of flour botanical sources as rice, oat, quinoa, buckwheat, sorghum, maize, and teff (Hager et al., 2012). Fiber has been considered an important nutritional aspect of foods, due to its role in human health. Thus, acorn fiber content can contribute to develop functional foods, additionally to the other acorn flour benefits. Insoluble fiber has also importance for baking performance since it increases water absorption and also swelling capacity, which impacts in rheological properties, leading to a decrease of viscosity and consequently usually reduces bread volume and increases bread's firmness (Föste et al., 2020; Sciarini et al., 2017). Particularly, for GF bread formulation, depending on the type of fiber, if well balanced, its addition to the formulation can develop further positive health effects such as glycemic index decrease, without reducing protein digestibility (Matos Segura and Rosell, 2011).

Regarding starch content, acorn flour presented about 50 g 100 g^−1^ DW, in accordance with other authors who also have studied starch, obtaining 51.79 ± 1.35 g 100 g^−1^ DW (Silva et al., 2016), and between 53.21 and 61.48 g 100 g^−1^ DW (López-Hidalgo et al., 2021). From the baking point of view, starch content is also another important flour's characteristic, since its rheological behaviour depend on starch amount and type. Thus, when using acorn flour in GF bakery, all these aspects are even more relevant, in a perspective of gluten replacing, since the interactions between starch, proteins, fiber, fat, and water, among flours blend, are essential for this role (Betoret and Rosell, 2020).

**Fig. 2:**
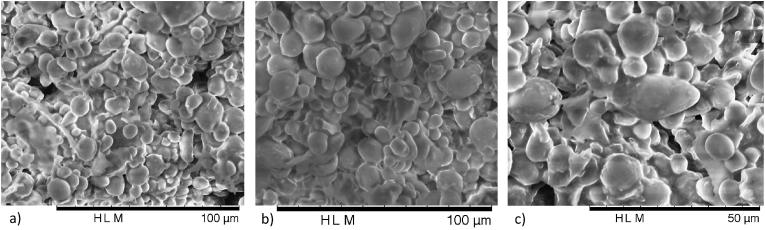
**–** Acorn flour SEM images with magnifying of: a) 800x, b) 1000x, and c) 1500x.

In Fig. 2 it is possible to observe images from SEM. It is very well perceived the spherical shape of acorn flour starch granules. The shape of starch granules is referred as important in its hydration properties, which as previously mentioned have a major importance in baking properties. When comparing with other botanical sources, which starch has a different structure, it is possible to understand this influence (Hager et al., 2012).

Starch structure can also affect CO_2_ volume produced by yeasts during fermentation, which also will have a major impact in bread characteristics (Sciarini et al., 2017; Ziobro et al., 2012). Acorn starch images from SEM has been previously presented, comparing fresh and dried acorn flours, and pointing out higher trend in *Quercus rotundifolia* to present damage starch, nevertheless it is great dependent on the process of flour milling (Correia, Leitão, & Beirão-Da-Costa, 2009). Moreover, starch granules microstructure is an important feature to better understand the behaviour of this flour as a baking ingredient (Betoret and Rosell, 2020; Matos and Rosell, 2015).

### 3.6 Pasting properties

Flours pasting properties are an additional important characterization parameter. For bakery purposes, especially in GF bread, where gluten replacement is based on starch properties, in particular gelatinization and retrogradation. As mentioned in previous studies, the contribution of each ingredient viscosity, according to its own profile is essential to the final product characteristics (Matos and Rosell, 2015). Amylose and lipid contents are one of the causes for the differences between the swelling capacity of starches (Singh et al., 2003). Acorn starch has about 50% amylose (Correia et al., 2013) and acorn is rich in fat content, so these two parameters are important for acorn flour pasting behaviour.

**Fig. 3:**
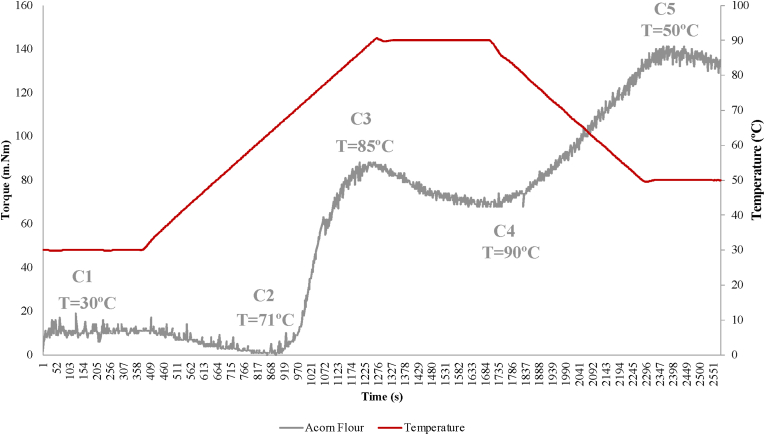
**–** Acorn flour pasting curve: Toque value (m.Nm) along time (s) and heating (°C).

In Fig. 3 it is possible to observe the pasting curve from acorn flour, where C1 represents maximum torque of mixing flours, C2 protein weakening, C3 starch gelatinization, C4 gel stability and amylase activity, and C5 starch retrogradation (Dang and Bason, 2015). The maximum torque (C1), was obtained with a constant temperature of 30*°*C revealing a torque value of 17 mN m. Next, minimum viscosity (C2), corresponding to protein weakening, occurred at 71 °C with a drop of torque value to 1 mN m. Following the heating process, viscosity increased until reaching the peak (C3), consistent with starch gelatinization at 85 °C, and a torque value of 87 mN m. This higher viscosity corresponds to a maximum of swollen-intact starch granules, that with temperature increase, and continuously water absorption, granules achieve its rupture point, and viscosity starts decreasing until reach a minimum (C4), which occurred at 90 °C showing a torque value of 68 mN m. Breakdown viscosity is the difference between C3 and C4 (19 mN.m), which represents the resistance to heat and shear. In other words, when breakdown viscosity is lower, means a higher starch granules resistance to heat and shearing stress. Finally, the test finished when temperature cools (50 °C), with amylose retrogradation, and reaching the highest torque value of 129 m.Nm, which supports predicting final product texture characteristics. Setback is the difference between C5 and C4 (61 mN.m), and it is an indicator of starch ability to undergo retrogradation.

Acorn starch structure has been associated with fewer amylose leaching and lower degree of gelatinization, and also its gelatinization higher temperature showed an increase resistance to swelling in comparison with other flours. Moreover, a lower gelatinization viscosity reveals a reduced swelling capacity as well (Correia et al., 2013). According to Correia et al. (2013), acorn presents high pasting temperature, showing high resistance to swelling and its starch is associated with a coherent structure. Nevertheless, a research conducted with *Quercus rotundifolia* from Algeria (Boukhelkhal and Moulai-Mostefa, 2017), presented lower amylose content (about 20%), although gelatinization temperature range (71.0–88.1 °C) was in accordance with the one obtained in the present work (85 °C). Additionally, Correia et al. (2013) referred that high consistency immediately after cooling suggest a great potential of acorn flours use in food industry and other industries.

## 4 Conclusions

Acorn flour is an ingredient presenting good nutritional and functional characteristics to recover its use in human nutrition, in particular in bakery products, with potential to many other uses. Acorn flour presents a high fat content, particularly monounsaturated and polyunsaturated, with high antioxidant capacity, although its effectiveness in the final product should be evaluated according to each formulation and purpose, as well as bioavailability and bioaccessibility. In regards to technological aptitude, acorn flour has a high amount of fiber mainly insoluble, and presents a starch content approximately of 50%. Its pasting behaviour reveals a high gelatinization temperature and resistance to heat and shear stress, showing that acorn flour could have an important contribution to texture and structure, depending on the type of product developed. Thus, the high amount of acorns that are being wasted, have the potential to be a valuable ingredient, with many uses and applications in human nutrition, giving a new value to this resource. However this flour has been consumed in human diet for many centuries, further investigation about anti-nutrients would be recommended in a future research, in order to analyze if eventually any negative effects of acorn flour could be found.

## CRediT authorship contribution statement

**Rita Beltrão Martins:** Conceptualization, Validation, Formal analysis, Investigation, Methodology, Data curation, Writing – original draft, Writing – review & editing. **Irene Gouvinhas:** Conceptualization, Methodology, Validation, Formal analysis, Investigation, Writing – review & editing, Supervision. **Maria Cristiana Nunes:** Conceptualization, Methodology, Validation, Formal analysis, Investigation, Writing – review & editing, Supervision. **Luís Mendes Ferreira:** Methodology, Validation, Formal analysis, Investigation, Resources, Writing – review & editing, Supervision, Funding acquisition. **José A. Peres:** Resources, Writing – review & editing, Supervision, Project administration, Funding acquisition, All authors have read and agreed to the published version of the manuscript. **Anabela Raymundo:** Conceptualization, Methodology, Validation, Formal analysis, Investigation, Resources, Writing – review & editing, Supervision, Project administration, Funding acquisition. **Ana I.R.N.A. Barros:** Conceptualization, Validation, Formal analysis, Formal analysis, Investigation, Resources, Writing – review & editing, Supervision, Project administration, Funding acquisition.

## Declaration of competing interest

The authors declare that they have no known competing financial interests or personal relationships that could have appeared to influence the work reported in this paper.

## Data Availability

The authors do not have permission to share data.
